# Linking Comparative Genomics of Nine Potato-Associated *Pseudomonas* Isolates With Their Differing Biocontrol Potential Against Late Blight

**DOI:** 10.3389/fmicb.2020.00857

**Published:** 2020-04-30

**Authors:** Mout De Vrieze, Adithi R. Varadarajan, Kerstin Schneeberger, Aurélien Bailly, Rudolf P. Rohr, Christian H. Ahrens, Laure Weisskopf

**Affiliations:** ^1^Department of Biology, University of Fribourg, Fribourg, Switzerland; ^2^Agroscope, Research Group Molecular Diagnostics, Genomics and Bioinformatics & SIB Swiss Institute of Bioinformatics, Wädenswil, Switzerland; ^3^Department of Plant and Microbial Biology, University of Zurich, Zurich, Switzerland

**Keywords:** *de novo* genome assembly, comparative genomics, biocontrol, *Pseudomonas*, phyllosphere, rhizosphere, beneficial microorganisms, genotype-phenotype correlation

## Abstract

For plants, the advantages of associating with beneficial bacteria include plant growth promotion, reduction of abiotic and biotic stresses and enhanced protection against various pests and diseases. Beneficial bacteria rightly equipped for successful plant colonization and showing antagonistic activity toward plant pathogens seem to be actively recruited by plants. To gain more insights into the genetic determinants responsible for plant colonization and antagonistic activities, we first sequenced and *de novo* assembled the complete genomes of nine *Pseudomonas* strains that had exhibited varying antagonistic potential against the notorious oomycete *Phytophthora infestans*, placed them into the phylogenomic context of known *Pseudomonas* biocontrol strains and carried out a comparative genomic analysis to define core, accessory (i.e., genes found in two or more, but not all strains) and unique genes. Next, we assessed the colonizing abilities of these strains and used bioassays to characterize their inhibitory effects against different stages of *P. infestans*’ lifecycle. The phenotype data were then correlated with genotype information, assessing over three hundred genes encoding known factors for plant colonization and antimicrobial activity as well as secondary metabolite biosynthesis clusters predicted by antiSMASH. All strains harbored genes required for successful plant colonization but also distinct arsenals of antimicrobial compounds. We identified genes coding for phenazine, hydrogen cyanide, 2-hexyl, 5-propyl resorcinol and pyrrolnitrin synthesis, as well as various siderophores, pyocins and type VI secretion systems. Additionally, the comparative genomic analysis revealed about a hundred accessory genes putatively involved in anti-*Phytophthora* activity, including a type II secretion system (T2SS), several peptidases and a toxin. Transcriptomic studies and mutagenesis are needed to further investigate the putative involvement of the novel candidate genes and to identify the various mechanisms involved in the inhibition of *P. infestans* by different *Pseudomonas* strains.

## Introduction

*Pseudomonas* bacteria are members of a genetically and phenotypically diverse genus of Gammaproteobacteria, famous for their widespread distribution across habitats as broad as water, soil, plants, animals, and humans. Plant-associated *Pseudomonas* show remarkable metabolic polyvalence, ensuring fitness and competitiveness in the rhizo- and phyllosphere, where they develop pathogenic, commensal or mutualistic relationships with their hosts (Clarke, 1982; Silby et al., 2011). Beneficial effects of *Pseudomonas* species on plants include plant growth promotion through facilitated uptake of nutrients and enhanced root growth, and higher resilience against abiotic and biotic stress, e.g., by interfering with phytohormone levels (van Loon, 2007; Lugtenberg and Kamilova, 2009; Hayat et al., 2010). Several strains, mainly belonging to the species *P. protegens*, *P. chlororaphis*, *P. fluorescens*, and *P. putida*, have been studied for their potential use as antagonists against insect pests and plant diseases caused by fungi, bacteria and oomycetes (Howell and Stipanovic, 1979; Thomashow and Weller, 1988; Keel et al., 1992; Maurhofer et al., 1994; Larkin and Fravel, 2001; Hultberg et al., 2010; Stockwell et al., 2010; Mehnaz et al., 2014; Arseneault et al., 2016; Caulier et al., 2018; Zengerer et al., 2018). These studies have highlighted various mechanisms by which plant-associated *Pseudomonas* can potentially protect plants from pathogens and pests, including (i) direct antibiosis by means of the production of secondary metabolites such as antibiotics, biosurfactants and toxins, (ii) niche occupation and competition, e.g., through siderophore-mediated iron uptake, and (iii) induction of plant defense mechanisms, especially induced systemic resistance (ISR) conferring broad-range protection against plant pathogens (van Loon et al., 1998; Ramamoorthy et al., 2001; Pieterse et al., 2014). Secondary metabolites such as cyclic lipopeptides, phenazines and other antifungal compounds, siderophores, or bacterial secretion systems and associated toxins and effectors, play an important role in the activity of antagonistic bacteria against plant pathogens. In previous work, a *Pseudomonas* strain collection was isolated from field grown potato plants and screened for antagonistic activity against the notorious potato pathogen *Phytophthora infestans* (Hunziker et al., 2015), an extremely potent hemibiotrophic oomycete and causal agent of late blight disease (Fry, 2008). Some of these *Pseudomonas* strains caused a strong reduction of pathogen growth *in vitro* both through emission of volatile compounds (De Vrieze et al., 2015; Hunziker et al., 2015) and through diffusion of non-volatile metabolites (Guyer et al., 2015; Hunziker et al., 2015). Moreover, significant reduction of disease symptoms was observed on leaf discs treated with single (Guyer et al., 2015) or combinations of (De Vrieze et al., 2018) the *Pseudomonas* strains. This anti-*Phytophthora* activity was consistently observed against a collection of genetically diverse lineages of *P. infestans* (De Vrieze et al., 2019). Despite these promising antagonistic activities, the underlying mechanisms explaining both *in vitro* and *in vivo* anti-*Phytophthora* activities of the various *Pseudomonas* strains had not yet been investigated.

The aim of the present study was to unravel the genetic determinants possibly involved in the antagonistic activity of our most active *Pseudomonas* strains by pairing comparative genomics and genome mining approaches to detailed anti-*Phytophthora* activity profiling of the strains. As fragmented, short read-based genome assemblies of widely used *Pseudomonas* reference strains can miss important genes encoding non-ribosomal peptide synthetases (NRPS) and phenazine biosynthesis genes (Varadarajan et al., 2020), we *de novo* assembled complete, contiguous genomes for nine strains of varying antagonistic potential. The effects of the nine selected *Pseudomonas* strains on *P. infestans* were further characterized by means of bioassays targeting different key stages of the pathogen’s lifecycle. The obtained phenotypic characteristics were then correlated to the genotype information, in a first step by assessing a literature-derived list of genes coding for known antibiotics, siderophores, cyclic lipopeptides, toxins, secretion systems and compounds involved in bacterial competition and plant-bacteria communication. In a second step, accessory genes obtained from comparative genomics analysis were correlated with assigned phenotypic subgroups of active versus inactive strains. This allowed identifying new candidate genes putatively involved in the various anti-oomycete activities displayed by the potato-associated *Pseudomonas.*

## Materials and Methods

### Bacterial Strains, Culture Conditions, and DNA Isolation

Nine bacterial strains previously isolated from the roots (R) and shoots (S) of field grown potato plants (Hunziker et al., 2015) were selected based on their activity against *P. infestans in vitro* (Guyer et al., 2015; Hunziker et al., 2015) and their origin (rhizosphere versus phyllosphere). The strains were kept at −80°C in 25% glycerol for long-term storage and plated on Lysogeny Broth (LB) medium for DNA extraction and biological assays. LB medium was prepared by dissolving 20 g/l of Difco LB broth (Lennox, United States) supplemented with 15 g/l of agar (Agar-agar, ERNE surface AG, Switzerland). For colonization assays on plants, rifampicin resistant strains were used, which were obtained as described previously (Guyer et al., 2015). Bacterial suspensions were prepared by suspending two to three bacterial colonies from 2 to 3 days old in NaCl (0.9%) and by spreading 50 μl of these suspensions on fresh LB plates. After 24 h, the newly grown bacterial cells were washed off the LB plates and suspended in NaCl (0.9%). For isolation of the rifampicin resistant strains from plants, selective cetrimide nalidixic acid (CN) medium for *Pseudomonas* was used (Biokar, France). High quality genomic DNA (gDNA) was prepared from overnight cultures grown in liquid LB medium using the GeneElute Bacterial DNA kit (Sigma-Aldrich, United States).

### Genome Sequencing, Comparison, Functional Annotation, and Mining

#### Genome Sequencing, Assembly, and Annotation

For each strain, an insert library was prepared from the gDNA, size selected with BluePippin (10 kb) and sequenced on Pacific Biosciences’ (PacBio) RSII sequencing platform (one SMRT cell per strain; P6-C4 chemistry). Initial quality control of reads, pre-assembly steps and *de novo* genome assembly were done as described previously (Omasits et al., 2017), either using HGAP3 (Chin et al., 2013) or ABruijn assembler (Lin et al., 2016) for *de novo* genome assembly. Subsequently, terminal repeats were removed, the contigs circularized, the start position adjusted to the *dnaA* gene and raw fastq Illumina reads (all strains were also sequenced using Illumina MiSeq; 2 × 300 bp paired end reads) were mapped to the respective PacBio contigs to correct indels in homopolymer regions, as described previously (Schmid et al., 2018a). Non-mapping Illumina MiSeq reads were *de novo* assembled using SPAdes (Bankevich et al., 2012) to identify potential plasmids. The final genome sequences were annotated with the Prokaryotic Genome Annotation Pipeline (v 4.0) of the National Center for Biotechnology Information (NCBI) (Tatusova et al., 2016) and are described in Supplementary Table S1. A further functional gene or protein annotation was carried out using several tools including eggNOG-mapper (v 0.12.7) and interproscan as described previously (Fernández et al., 2019), considering hits with an *E*-value below 0.001. The COG (Cluster of Orthologous Groups) categories were extracted in an effort to link functions with data from phenotypic assays. To identify secondary metabolite biosynthesis gene clusters, antiSMASH (v 4) was run on the protein sequences (Blin et al., 2017) (toggles on: ClusterFinder, BGC border prediction, all the extra features) without restriction on cluster size and minimum number of biosynthesis-related PFAM domains; the minimum ClusterFinder probability was set to 50%. The output was parsed with a custom python script.

#### Phylogenetic analysis

A phylogenetic tree was computed with the nine genomes and those of 48 other selected *Pseudomonas* species (downloaded from NCBI’s RefSeq), covering a broad range of lineages and subgroups in the *Pseudomonas* genus; *Azotobacter vinelandii* DJ was used as outgroup (Supplementary Table S2). For this, protein coding gene sequences of 83 essential core genes present in all 58 genomes were used as input for the software bcgTree (Ankenbrand and Keller, 2016); the final tree was bootstrapped 100 times in RAxML (Stamatakis, 2014) and visualized with Figtree^1^.

#### Comparative Genomics: Analysis of Pan, Core, Accessory, and Unique Genes

Genomic features of the nine strains were compared to define pan, core, accessory and unique coding sequences (CDS), i.e., protein-coding genes, using the software pipeline Roary (v 3.7.0) (Page et al., 2015) as described (Schmid et al., 2018b). Of 51,093 proteins, 38 were too short (<120 bp), and thus not included in the analysis. Additionally, a separate comparative analysis was performed using R32 as the reference sequence and querying all its protein coding sequences against the eight other genomes using the Blast Atlas module of Gview Server (Petkau et al., 2010) (*E*-value cut-off of 1e-5; amino acid identity cut-off of 50%).

### Phenotypic Assays

The inhibitory activity of the bacterial strains against *P. infestans* was characterized with four *in vitro* assays targeting (i) mycelial growth, (ii) sporangia germination, (iii) zoospore release, and (iv) zoospore germination. Additionally, the plant colonization performance of the bacterial strains was evaluated in a greenhouse trial. *P. infestans* was routinely maintained on rye agar supplemented with D-glucose (5g/l) or on pea agar. Rye glucose agar (further referred to as rye agar) was prepared by simmering 200 g of rye grains for 1 h in 1 l of nanopure water. Using cheese cloth, the rye grains were filtered out and 15 g/l of agar and 5 g/l of D-glucose were added to the broth before autoclaving. Pea agar was prepared by autoclaving 200 g of frozen peas in one liter of nanopure water. After filtration, 15 g/l of agar was added to the liquid, which was autoclaved a second time. The oomycete was regularly passaged on potato slices in between subculturing on rye agar plates to maintain its virulence.

#### Mycelial Growth

Bacterial strains and *P. infestans* were co-inoculated on rye agar plates. One 5 mm rye agar plug of a 14 days old *P. infestans* plate was placed in the centre of the plates whereas three drops of 10 μl of bacterial suspensions (OD_570_ = 1) were pipetted at the margins of the plate. The plates were incubated in the dark for 6 days at 18°C before being photographed. Mycelium growth was assessed by measuring the growth area on the pictures using the software ImageJ as described previously (De Vrieze et al., 2015). The experiment was repeated once, each repetition containing four technical replicates.

#### Sporangia Germination

Sporangia suspensions were prepared by scraping mycelium off 14 days old plates and suspending it in demineralized water. After vigorous shaking, the suspension was filtered using cloth in order to discard the mycelium. Concentration was determined using a Thoma chamber and adjusted to 200,000 sporangia/ml. Sporangia suspensions were maintained in the dark until use. Bacterial suspensions (OD_570_ = 2) were prepared and mixed one to one with the sporangia suspension. Of these mixtures, 15 μl were spotted on water agar plates. In total, four plates with four drops were prepared and incubated in the dark at 18°C. Pictures were taken after 24 h using a binocular camera (Leica). Using ImageJ, numbers of germinated sporangia and atypically germinated sporangia were counted and categorized according to their phenotype. Germ tubes of regularly and atypically germinating sporangia combined were highlighted on the pictures using MS Paint and mean germ tube length was estimated using a macro-instruction in ImageJ. The experiment was conducted twice and contained a minimum of 3 (treatments) to 6 (control) technical replicates per repetition, on average 333 sporangia were counted for each replicate.

#### Zoospore Release

Sporangia suspensions (100 μl, as prepared above) were mixed in a one to one ratio with bacterial suspensions (OD_570_ = 2) in 1.5 ml Eppendorf tubes. To trigger zoospore release, 500 μl of ice-cold Modified Petri’s solution (5 mM CaCl2, 1 mM MgSO4, 1 mM KH2PO4 and 0.8 mM KCl) were added to the mixtures (Petri et al., 1917). The tubes were stored at 4°C for 2 h and then left to rest at room temperature for 20 min. The suspensions were thoroughly mixed and 30 μl drops were spotted onto a 24-well plate. One well contained one treatment and the experiment was conducted three times. Pictures were taken at fourfold magnification using a Cytation5 plate reader (Biotek, United States).

#### Zoospore Germination

For zoospore germination, zoospore suspensions were obtained by adding ice-cold Modified Petri’s solution to sporangia suspensions. After incubation times of 2 h at 4°C and 20 min at room temperature, 30 μl were carefully pipetted from the upper layers of the suspension in the tubes and spotted onto empty 24-well plates, as zoospores tend to swim toward the liquid surface. Mixing of the tubes was avoided to prevent pipetting sporangia rather than zoospores. Bacterial suspensions were added at a one to one ratio in the wells. One well per treatment and two to three control wells containing only zoospores were prepared. The plates were incubated at 15°C for 4 h. Pictures were taken at a 10-fold magnification using a Cytation5 plate reader (Biotek, United States) at the end of the incubation time. Germinated zoospores were counted and categorized according to their phenotype. On average, 129 zoospores were counted per picture. The experiment was repeated twice.

#### Epi- and Endophytic Plant Colonization

Potato tubers of cultivars Bintje, Lady Claire and Victoria were treated with bacterial suspensions as follows. Per strain, two potato tubers of each cultivar were placed in half-filled planting pots. Ten ml of rifampicin resistant bacterial suspensions (OD_570_ = 1) were pipetted onto the tubers. The pots were filled to the brim with potting soil immediately after treatment and placed in trays. The pots were further watered via the trays. Treatments were not mixed within one tray to avoid cross-contamination via the watering. Five weeks after planting, one stem per pot was harvested. After removal of the leaves, three portions of about 12 cm were cut from the base, center and top of the stems. Each portion was cut in half. The first halves were surface sterilized by submerging the cuttings in alcohol (70%) for 1 min. The cuttings were rinsed twice in distilled sterile water, and then left to air-dry for approximately 5 min. The second halves were not surface-sterilized. Three radial discs were cut in both the surface-sterilized and non-sterilized portions, avoiding the first centimeter from each extremity and with approximately one-centimeter distance between each radial disc. These stem cuts were placed on selective *Pseudomonas* CN media supplemented with rifampicin. After 5 days, bacterial growth originating from the stems cuts was assessed and counted for each stem height and each cultivar.

#### Data Analysis and Statistics

All statistical tests and analyses were performed using R software (R Core Team, 2008). Multiple comparisons between treatments were performed for the different experiments by using the Kruskal Wallis test followed by Fisher’s least significant difference for grouping from the R-package agricolae (de Mendiburu, 2017). The boxcox transformation in the MASS package (Venables and Ripley, 2002) was used for transformation of variables when needed. For graphical representation of sporangia germination, one repetition was selected. For the sporangia and zoospore germination assays, differences in treatments were visualized in a principal component analysis (PCA) using the packages FactoMineR (Le et al., 2008) and FactoExtra (Kassambara and Mundt, 2017). Germ tube length was added as a supplementary variable to the PCA for sporangia germination.

### Analysis of Known Determinants of Antimicrobial and Plant Colonizing Abilities Activity

Proteins encoded by 35 gene clusters (Supplementary Table S4) involved in the production of compounds of interest for biocontrol including antibiotics, cyclic lipopeptides, siderophores, exoenzymes, toxins, type III secretion effectors, and those involved in plant-bacterial communication and plant colonization (overall 319 proteins) were selected from the literature. Protein sequences were searched against the nine genomes using blastp (v 2.2.30 +) (Altschul et al., 1990). Only the best matching hit satisfying certain threshold parameters (*E*-value ≤ 1e-5, percent identity ≥ 50% and query coverage ≥ 50%) was considered as homolog (integrating also synteny information of the compared genes); otherwise, they were considered to be absent.

### Exploring Correlations Between Known Genetic Determinants and Phenotypes

The results of the inhibition and plant colonization assays were summarized in a phenotypic table. For the inhibition assays, the results were translated to percentages of inhibition in comparison to controls. For the colonization assays, only the assay on Lady Claire was retained and a colonization performance score was attributed to each strain, by calculating the percentage of the number of times the bacteria were successfully retrieved from the stem cuts over the total number of stem cuts made for the three different heights. Data on the inhibition of *P. infestans* development on potato leaf discs of cultivars Bintje, Lady Claire and Victoria was retrieved from a previous study (De Vrieze et al., 2018) and added to the phenotypic table. In these experiments, mixtures of sporangia and bacterial suspensions were pipetted on potato leaf discs. Redundancy analysis (RDA) was used to investigate the correlation between the known genetic determinants (considered as explanatory variables) and the observed phenotypes (considered as response variables). Prior to the RDA, clustering among the compounds was needed to reduce the number of variables and group genetic determinants according to their co-occurrences in the genomes of the bacterial strains. Only compounds for which complete gene clusters were found were integrated in the analysis. Compounds present or absent in all strains were left out. We chose the best clustering method following chapter 4 of Borcard et al. (2018). Then the RDA analysis was performed following chapter 6 of Borcard et al. (2018) and using the R package vegan (Oksanen et al., 2019). PCA analysis of the genetic determinants and of the observed phenotypes were also performed.

### Exploring Correlations Between Accessory Genes and Phenotypes

The “gene_presence_absence.csv” output file obtained from Roary was associated with the phenotypic table to explore the association of the accessory genes (genes shared among two or more of the nine genomes but not all) with selected observed traits (mycelial growth on pea agar medium, sporangia germination, germ tube development and colonization). For each assessed trait, the strains were categorized as “active” (value of 1) when exhibiting clear inhibitory activity or “non-active” (value of 0) when low or no activity was observed. Correlations were obtained using Scoary (with default parameters and *p-*value 0.05), a tool to study pan genome wide association studies (Brynildsrud et al., 2016).

## Results and Discussion

### Genomic Characterization, Phylogenetic Relationship, and Comparison of the Nine *Pseudomonas* Strains

The nine *Pseudomonas* strains were sequenced, and *de novo* assembled to create high quality, complete reference genomes. Each genome was assembled into a single chromosome, except for strain R76, which contained one additional plasmid (7.2 kb; Table 1). The genome size varied between 5.65 and 7.20 Mbp for R32 and R47, respectively, while the coverage ranged from 66-fold for S19 up to 160-fold for strain R32. Combined with the Illumina data, genome coverage exceeded 160-fold for all strains. A repeat analysis revealed that nearly identical repeat sequences of up to ∼35 kilobase pairs were present (Schmid et al., 2018a), which could only be successfully resolved by using long PacBio reads. The complete genomes allowed to identify comprehensive gene inventories, including all genes relevant for antibiosis (Varadarajan et al., 2020). They also represented the best basis for a comparative genomics analysis, which can miss core genes when fragmented short read-based assemblies are compared (Schmid et al., 2018b). The genome properties are listed in Table 1; the list of genes from each of the nine genomes including their annotation, COG categories and additional functional information from interproscan and eggNOG is provided in Supplementary Table S3.

**TABLE 1:** Overview of genome characteristics of the nine *Pseudomonas* strains sequenced in this study.

**Feature**	**R32**	**R47**	**R76**	**R84**	**S04**	**S19**	**S34**	**S35**	**S49**
Genbank accession #	CP019396	CP019399	CP019428	CP019426	CP019427	CP019397	CP019398	CP019431	CP019432
# Chromosomes	1	1	1	1	1	1	1	1	1
Size (Mbp)	5.65	7.20	6.82	6.60	6.10	6.10	6.27	6.61	6.66
Plasmid size (kbp)	–	–	7.2	–	–	–	–	–	–
Coverage (PacBio)	160	92	117	102	86	66	121	114	114
Coverage (MiSeq)	106	73	87	107	112	109	90	106	111
Longest repeat (nt)	12,077	7535	7540	7797	17,803	17,803	6071	25,099	35,198
% G + C	62	63	60	59	60	60	60	60	59
# Genes	5233	6519	6309	5936	5612	5614	5743	6032	5996
# CDS	5078	6315	5994	5757	5383	5385	5543	5822	5816
# Pseudogenes	58	116	226	83	136	136	103	116	84
# Average CDS length (aa)	331	326	327	333	329	329	326	334	332
Coding (%)	89	86	86	87	87	87	86	88	87
rRNA genes	19	16	16	19	22	22	22	19	19
tRNA genes	74	68	69	73	67	67	71	71	73
miscRNA	4	4	4	4	4	4	4	4	4

*Strains prefixed with (R) and (S) were isolated from roots and shoots of field grown potato plants respectively.*

**FIGURE 1:**
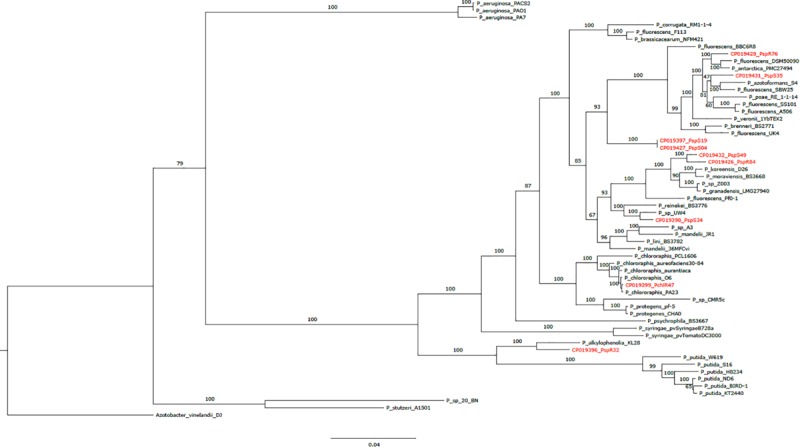
Phylogenetic tree based on 107 core genes from nine sequenced *Pseudomonas* strains (this study; marked in red), 48 *Pseudomonas* reference genomes and one outgroup (*Azotobacter vinelandii* DJ). Bootstrap values obtained from 100 bootstrap runs are shown for each node. The scale at the bottom indicates the number of amino acid substitutions per site.

We next inferred the phylogenetic and taxonomic relationship of the newly sequenced genomes in the context of 48 reference *Pseudomonas* genomes including known biocontrol strains based on a phylogenetic tree (Figure 1). High bootstrap support for the majority of branches indicated that the phylogenetic tree is quite robust. Furthermore, the tree closely resembled a previous lineage classification of *Pseudomonas* based on the analysis of four housekeeping genes from 107 strains (Mulet et al., 2010). All genomes except that of strain R32 fell into one of these groups/subgroups. R76 and S35 clustered with the *P. fluorescens* subgroup, as well as S04 and S19, which however formed a separate sub-clade (Figure 1). S49 and R84 clustered with the *P. koreensis subgroup*, while R47 clustered with the *P. chlororaphis* subgroup. We could not assign S34 to any specific subgroup. Notably, all of the above subgroups, which contained sequenced genomes except R32, belonged to the *P. fluorescens* group. Strains S04 and S19 fell in the same branch, reflecting their almost identical genome sequences (Table 1). We observed that R47 was closely related to already described biocontrol strains belonging to species such as *P. chlororaphis, P. chlororaphis sub. Aureofaciens*, and *P. protegens* (Kupferschmied et al., 2013; Flury et al., 2016). We also observed that strain R32 had the smallest of the nine sequenced genomes. This strain clustered together with *P. alkylophenolia* KL28 in a separate sub-clade along with the *P. putida* group (Figure 1).

**FIGURE 2:**
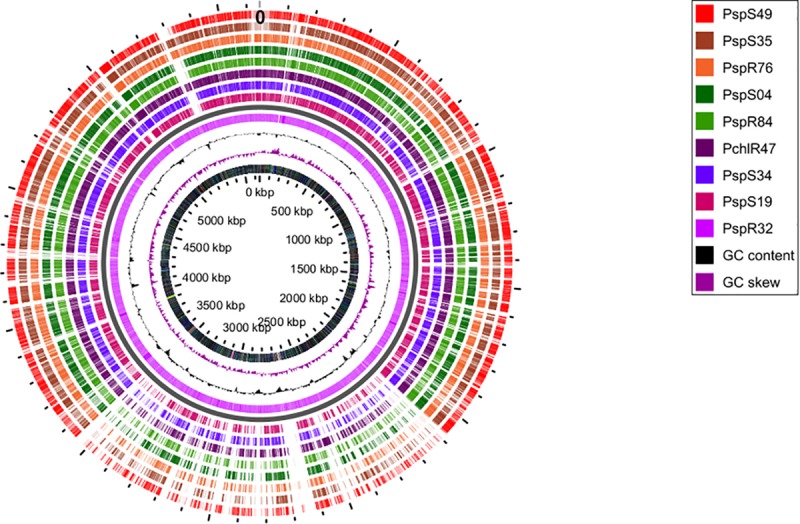
Circular genome view with R32 strain as reference. Moving inward, tracks 1–8 visualize the BLAST results (presence/absence) of all CDSs from each of the sequenced genomes, against the reference. Track9 (mauve) shows the CDS for R32. The tenth (black) and eleventh (purple) rings show the GC content and GC skew of the R32 genome respectively. The innermost ring shows the genomic coordinates of strain R32.

Finally, we obtained the core, pan and accessory genes based on a comparative genomics analysis of the nine sequenced strains. Roary predicted 2,860 core CDS clusters (28,610 genes), 4,694 unique genes clusters (4,899 genes), and 4,277 accessory gene clusters (present in a subset of two or more but not all strains; 17,547 genes). Table 2 shows the detailed results for these categories per genome. We finally compared all protein-coding sequences of the smallest genome (R32) against the other genomes (Figure 2) and observed that most of the genes that are unique to strain R32 were located near the terminus of replication. The occurrence of strain-specific genes near the terminus of replication has been associated with genome plasticity in several comparative genomics studies (Suyama and Bork, 2001; Read et al., 2003).

**TABLE 2:** Results of a comparative genomics analysis of nine *Pseudomonas* strains.

***Pseudomonas* strain**	**No. of core CDS**	**No. of accessory CDS**	**No. of unique CDS**	**No. CDS (<120 bps)**	**Total No. of CDS**
R32	3120	1200	754	4	5078
R47	3210	2129	972	4	6315
R76	3153	1914	923	4	5994
R84	3164	2210	380	3	5757
S04	3203	2175	0	5	5383
S19	3203	2176	1	5	5385
S34	3240	1671	628	4	5543
S35	3141	1826	850	5	5822
S49	3176	2246	390	4	5816

*Count of protein coding sequences (CDS) in core (CDS shared between all strains), accessory (CDS shared by two or more but not all strains) and unique (CDS unique to a particular strain) CDS clusters are given for each strain. CDS below 120bp were excluded from the analysis.*

### Genome Mining for Known Genetic Determinants Involved in Antimicrobial Activity and Plant Colonization

**FIGURE 3:**
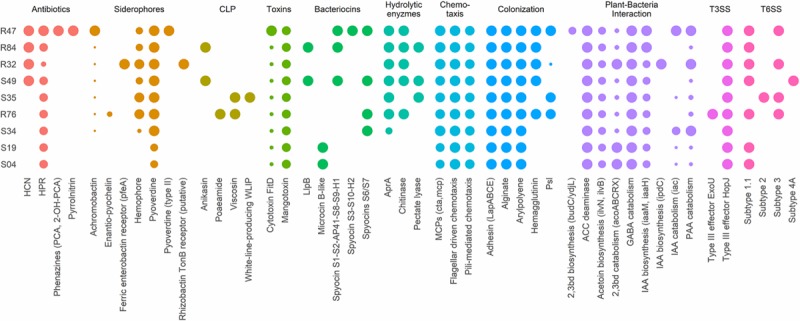
Presence/absence table of known antibiotics, siderophores, cyclic lipopeptides (CLP), toxins, bacteriocins, and extracellular hydrolases with reported antibacterial, antifungal or anti-oomycetal properties (2,3bd, 2,3-butanediol; PAA, phenylacetic acid; IAA, indole-3-acetic acid, GABA, γ-aminobutyric acid; ACC, 1-aminocyclopropane-1-carboxylic acid). All genomes were also screened for genes implicated in chemotaxis, plant colonization, plant-bacteria interactions, type III secretion systems (T3SS) and type VI secretion systems (T6SS). Full sized dots indicate the presence of homologs for all necessary genes or complete gene clusters. Small dots indicate incomplete clusters and dot size is proportional to the number of homologous genes found. All genes and reference sequences, including the genes encoding for traits for which no homologous genes were found, can be found in Supplementary Table S4. Strains are grouped according to their phenotypical activity *in vitro* (R47, R84, R32, and S49) and on plant tissue (R32, S49, S35). The least inhibiting strains are at the bottom of the table (S34, S19, S04).

Beneficial plant-associated microorganisms provide their hosts with enhanced protection against pathogenic invaders, by triggering plant immunity, by competing with pathogens for space, by impounding nutrients or by direct antibiosis. When confronted to a disease like *P. infestans*, which can be seed-, soil- or air-borne, both the ability to produce antimicrobial compounds affecting the pathogen’s different life stages and the ability to colonize and thrive in the plant’s rhizosphere and phyllosphere are important assets for biocontrol agents. We therefore considered these different phenotypes when searching for putative determinants within our compiled list of genes known to be involved in biocontrol activity (Figure 3).

**FIGURE 4:**
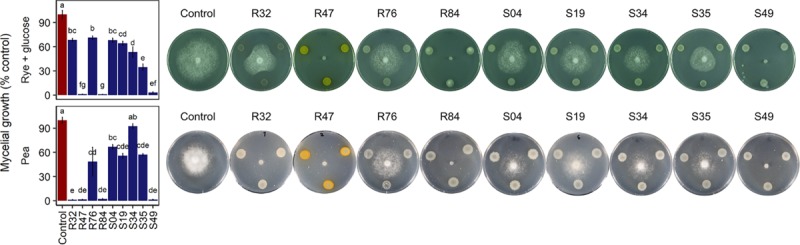
Effect of *Pseudomonas* strains on mycelial growth on rye glucose agar **(top)** and pea agar **(bottom)** after 7 days of incubation. Growth is expressed as percentage of the corresponding unexposed controls. Letters indicate significant differences between the strains and the unexposed control (Kruskal–Wallis’ test, *p* < 0.05; see section Materials and Methods).

*P. infestans* spreads in plant tissue by producing multinucleate hyphae that form an invasive network of mycelium in the plant’s intracellular space (Fry, 2008). For dispersal, *P. infestans* produces arborescent mycelial structures, the sporangiophores, which carry large spores called sporangia. Upon maturity, the sporangia detach from the sporangiophores and are dispersed by water and wind. Depending on temperature and humidity, sporangia either directly germinate and infect new plant tissue or they release motile zoospores (Fry, 2008). Zoospores can swim for several hours in order to find a suitable infection site, where they will encyst and readily produce an appressorium, from which a germ tube destined to penetrate plant tissue is formed. Mycelial growth, sporangia germination and zoospore release and germination thus represent key elements to the pathogen’s successful proliferation. Bacterial strains R32, R47, R84, and S49 completely inhibited mycelial growth of *P. infestans in vitro* on pea agar (Figure 4). We observed that all four active strains carried the *hcnABC* genes necessary for the production of hydrogen cyanide (HCN) (Figure 3), a bacterial volatile with broad antifungal activity (Voisard et al., 1989). The loss of activity of R32 on rye glucose agar medium could be explained by the medium dependency of HCN production and/or the reduced growth of R32 on this medium. However, the significant inhibition observed on both media for non-HCN-producers provides evidence of the production of other active compounds (Figure 4). The occurrence of genes coding for hydrolytic enzymes such as chitinase (R47, R84, R32, S49, R76) and exoprotease *aprA* (R47, R84, R32, S49, S35, and R76) in the strains causing strong mycelial inhibition and their absence from most strains not showing such activity (S34, S04, and S19) might indicate a possible role of either or both enzymes in the inhibition of *P. infestans* (Figure 3). For the exoprotease *aprA*, activity against nematodes was reported (Siddiqui et al., 2005), whereas chitinases have been shown to be involved in *Pseudomonas* mediated biocontrol of plant pathogenic fungi (Koby et al., 1994; Nielsen et al., 1998). However, unlike fungi and nematodes, oomycetes such as *P. infestans* do not contain chitin; yet, plants have been shown to respond to oomycete infection by upregulating chitinases, suggesting a possible role of these enzymes in defense against oomycetes too (Hardham, 2007). In addition to these enzymes, the dark orange pigmentation of R47 (Figure 4) bears witness of hydroxyphenazine (2-OH-PCA) production, a derivate of the phenazine 1-carboxylic acid (PCA). *Pseudomonas* commonly produce two or more different types of phenazines, which display an extensive spectrum of activity against both Prokaryotes and Eukaryotes (Laursen and Nielsen, 2004; Mavrodi et al., 2006; Zengerer et al., 2018). The production of PCA by *Pseudomonas fluorescens* LBUM636 was shown to be involved in the inhibition of mycelial growth of *P. infestans in vitro* and on potato tubers (Morrison et al., 2016). Another study postulated that the different forms of phenazines determine the biocontrol spectrum of antagonistic strains and highlighted the higher potency of 2-OH-PCA to inhibit the oomycete *Pythium ultimum* when compared to PCA (Yu et al., 2018). Yet, apart from R47, none of the other mycelium-inhibiting strains were identified as phenazine producers, suggesting that phenazines are not the sole determinants of this anti-*Phytophthora* activity. The same observation is true for other antibiotic gene clusters encoded in the R47 genome, e.g., HPR and pyrrolnitrin (Figure 3). While these compounds might contribute to the observed mycelial inhibition, their absence from non-cyanogenic strains which also did not produce phenazines but still displayed anti-mycelial activity suggest that other, yet unknown compounds or mechanisms are involved this inhibition of *P. infestans* mycelial growth.

**FIGURE 5:**
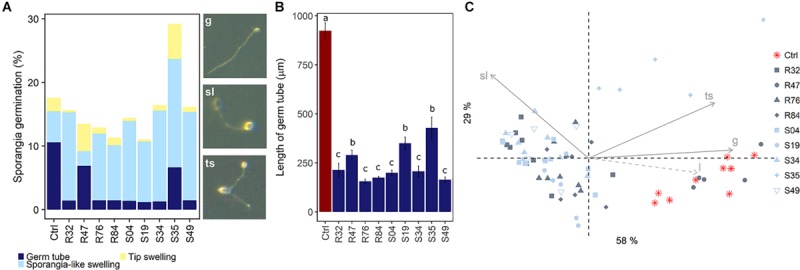
Effects of bacterial strains on sporangia germination. **(A)** Observed germination phenotypes included regular germination (g), sporangia-like swellings (sl) and tip-swelling (ts). **(B)** Mean sporangia germ tube length was estimated. Different letters indicate significant differences between strains (Kruskal Wallis’ test, *p* < 0.05; see section Materials and Methods). **(C)** PCA of sporangia germination as a function of germ tube phenotype. Germ tube length (l) was added as a supplementary variable (dashed arrow).

**FIGURE 6:**
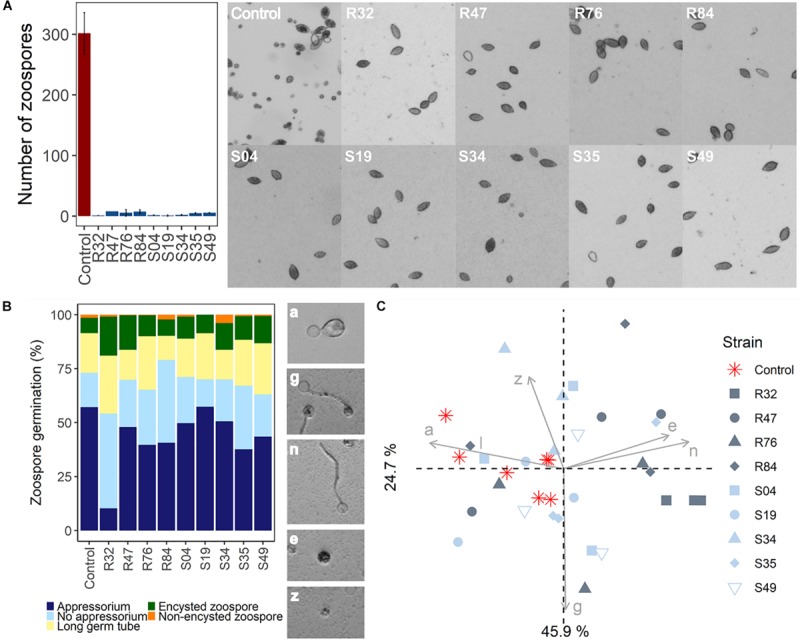
**(A)** Effects of bacterial strains on zoospore release. **(B)** Effects of *Pseudomonas* strains on zoospore germination. Observed germination phenotypes included regular appressorium formation (a), long germ tube formation with appressorium (g), germ tube formation without appressorium (n), zoospore encystment (e), and non-encysted zoospore (z). **(C)** PCA of zoospore germination in function of germination phenotype.

In contrast to mycelial growth inhibition, the interference of the strains with other developmental stages of *P. infestans* are likely independent of the ability to emit HCN, since cyanogenic strains were not more active than non-cyanogenic ones (Figures 5, 6). Sporangia germination rates were generally lower when the sporangia were exposed to the bacterial strains, except for S35 (Figure 5A), while germ tube length was reduced by all strains (Figure 5B). The lowest germination rates were observed for R84 and S19 but the formation of the typical long germ tubes (Figure 5A) was drastically reduced in all strains but S35 and R47. All strains almost completely prevented sporangia from releasing zoospores, but only R32, R47, and S34 were able to also reduce the germination of zoospores released prior to strain exposure (Figure 6B). Zoospores exposed to R32 showed a remarkably higher proportion of germinating zoospores lacking appressoria. Interestingly, we even observed zoospore lysis upon exposure to R32 (Supplementary Figure S1). Different studies reported that the zoospores of *P. infestans* were lysed when exposed to high concentrations of cyclic lipopeptides like poaeamide, orfamide and massetolide, while immobilization and encystment of zoospores (preventing autoaggregation) were induced at low concentrations (Bruijn et al., 2007; Tran et al., 2007; Zachow et al., 2015). Cyclic lipopeptides (CLP’s) play important roles in *Pseudomonas* species, e.g., for biofilm formation, bacterial motility, virulence or antagonism against bacteria, fungi, oomycetes and protozoa (Raaijmakers et al., 2006, 2010). Although no CLP-like NRPS clusters were identified in R32, we detected such clusters in four strains showing reduced appressorium formation (Figure 6B), namely R84, S49, S35, and R76 (Table 3). A biosynthetic gene cluster for the cyclic lipopeptide anikasin was found in strains R84 and S49. A previous study by Götze et al. (2017) reported that anikasin isolated from *P. fluorescens* is required for swarming motility and has antagonistic activity against protozoal ameba present in the soil but did not find evidence for any antifungal activity against various fungi. Whether oomycetes such as *P. infestans* are susceptible to anikasin, or whether anikasin contributes to the inhibitory activity of these two *Pseudomonas* strains on *P. infestans* remains to be investigated. Viscosin producing clusters were identified in the two other active strains S35 and R76. Acebo-Guerrero et al. (2015) reported that three rhizosphere *P. chlororaphis* strains producing the biosurfactant viscosin showed biocontrol activity against *Phytophthora palmivora*. Additionally, we found a lipopeptide cluster encoding for poaeamide in R76 strain and WLIP (white-line-producing) in S35 strain (Table 3). While the reduction of appressoria formation was stronger for these four putative CLP producing strains than for the other strains investigated, the highest inhibition of zoospore germination was observed for R32, which, in addition to the observed zoospore lysis, suggests that yet to be identified CLPs or other compounds might have inhibitory potential against *P. infestans* zoospores (Figure 6). Taking together mycelium, sporangia and zoospore life stages, R32 stands out as the most effective inhibitor of *P. infestans in vitro*. Despite its smaller genome, iron acquisition related genes coding for siderophores and iron acquisition related compounds were more numerous in R32 than in the other strains (Figure 3). Strains R32, R47, R76, S34, S35, and S49 harbored genes for the production of pyoverdine. Pyoverdines in *Pseudomonas* strains have been linked to iron chelation, but also to the strains’ ability to colonize plant roots and survive in the soil. The genome of R32 however also encoded a receptor for ferric-enterobactin (*pfeA*) and a homolog for a putative TonB-dependent receptor for rhizobactin from *P. protegens* CHA0 was found in R32 as well (Jousset et al., 2014; Figure 3), suggesting that iron competition could be one of the mechanisms used by this *Pseudomonas* to inhibit different stages of *P. infestans* development, in addition to its likely role in rhizosphere competence and plant colonization (see below).

**TABLE 3:** Biosynthetic gene clusters as predicted by antiSMASH in the nine *Pseudomonas* genomes are shown here.

	**Region**	**Type**	**From**	**To**	**Most similar known cluster**
R32	Region 1	NRPS-like	108,600	137,959	Mangotoxin
	Region 2	arylpolyene	301,125	344,738	APE Vf
	Region 3	NRPS	1,763,102	1,815,679	Pyoverdine
	Region 4	NRPS	3,581,365	3,648,859	Pyoverdine
R47	Region 1	NRPS-like	114,171	144,819	Mangotoxin
	Region 2	arylpolyene	499,588	543,208	APE Vf
	Region 3	other	4,075,994	4,117,076	Pyrrolnitrin
	Region 4	NRPS, resorcinol	4,846,097	4,925,365	Pyoverdine
	Region 5	NRPS	4,969,815	5,022,843	Pyoverdine
R76	Region 1	NRPS-like	159,850	202,848	Mangotoxin
	Region 2	arylpolyene	525,506	569,081	APE Vf
	Region 3	NRPS	2,841,936	2,945,551	Viscosin
	Region 4	NRPS	3,563,564	3,604,450	Pyochelin
	Region 5	NRPS	4,364,529	4,407,587	Poaeamide
	Region 6	NRPS	4,766,660	4,819,556	Pyoverdine
R84	Region 2	NRPS-like	131,789	161,456	Mangotoxin
	Region 3	arylpolyene	499,287	542,891	APE Vf
	Region 4	NRPS, terpene	2,169,525	2,234,015	Pyoverdine
	Region 5	NRPS	2,763,610	2,840,351	Anikasin
	Region 6	NRPS	4,533,076	4,586,074	Pyoverdine
S04	Region 1	arylpolyene	423,375	467,009	APE Vf
	Region 2	NRPS	2,828,784	2,901,359	Pyoverdine
	Region 3	terpene	4,329,654	4,350,538	Pyoverdine
S19	Region 1	arylpolyene	423,375	467,009	APE Vf
	Region 2	NRPS	2,828,790	2,901,365	Pyoverdine
	Region 3	terpene	4,329,659	4,350,543	Pyoverdine
S34	Region 1	NRPS-like	96,203	125,383	Mangotoxin
	Region 2	arylpolyene	431,851	475,456	APE Vf
	Region 3	NRPS	4,344,492	4,408,473	Pyoverdine
	Region 4	NRPS	4,455,540	4,508,538	Pyoverdine
S35	Region 1	NRPS-like	109,236	150,641	Mangotoxin
	Region 2	arylpolyene	474,763	518,338	APE Vf
	Region 3	terpene, NRPS	2,731,917	2,858,142	Viscosin
	Region 4	NRPS	4,185,638	4,230,966	WLIP
	Region 5	NRPS	4,576,688	4,629,596	Pyoverdine
S49	Region 1	NRPS-like	120,934	150,633	Mangotoxin
	Region 2	arylpolyene	473,010	516,614	APE Vf
	Region 3	NRPS	2,191,853	2,267,429	Pyoverdine
	Region 4	NRPS	3,906,071	3,982,443	Anikasin
	Region 5	NRPS	4,658,162	4,711,160	Pyoverdine

*For each region, the biosynthetic cluster type, the genomic boundaries (in bp) and the most similar known biosynthetic gene cluster are shown.*

**FIGURE 7:**
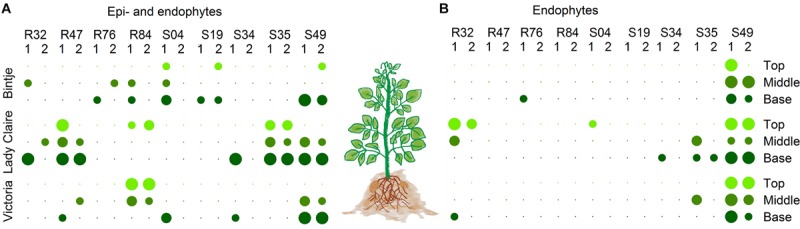
**(A)** Epiphytical and **(B)** endophytical colonization of the bacterial strains on tuber-treated plants after 4 weeks. For endophytical colonization, assessed stems were sterilized. Per cultivar, three stems cuts from at 3 different heights from stems of two different plants were taken. Dot color corresponds to the cut’s location on the plant (top, light green; middle, green; base, dark green). Dot size is proportional to the number of stems cuts out of which bacterial colonies grew (1–3).

Beyond the ability to inhibit a pathogen by direct antibiosis or competition, biological control agents need the capacity to colonize the host plant to provide efficient protection. Of all plant-colonizing bacteria, only a limited number are effective endophytical colonizers (Lindow and Brandl, 2003). By inoculating tubers and collecting stems at different heights, we assessed the performances of the strains to colonize potato plants epiphytically (Figure 7A) and endophytically (Figure 7B). This experiment was performed on three different potato cultivars, since plant genotype has been reported to strongly affect colonization by bacteria (Andreote et al., 2010; Costa et al., 2012). Bacterial strains were found at different heights in the plant. As expected, the strains were isolated more frequently from the non-sterilized stem cuts, revealing more frequent epiphytic than endophytic colonization. Lady Claire was clearly the preferred cultivar for epiphytic colonization by R47 and S35 (Figure 7A). Strain S49 on the other hand turned out to be the most potent endophytic colonizer with no cultivar preference (Figure 7B). R32 and S35 seemed able to establish endophytically in Lady Claire plants. R32’s genome encoded numerous plant hormone-related compounds (Figure 3), which could be of particular importance in rhizosphere competence as well as endophytical colonization. Interestingly, both S35 and R32, together with R76, differed from the other strains by carrying an incomplete gene cluster (*hasDEFIS*) homologuous to the hemophore-dependent iron acquisition biosynthetic cluster *hasADEFIRS* previously identified in *P. protegens*, *P. fluorescens*, *P. chlororaphis*, and *P. gessardii* strains (Garrido-Sanz et al., 2016). Bacteria use hemophores, which are extracellular proteins scavenging and delivering heme to specialized receptors, to acquire iron (Hartney et al., 2011). R76 and S35 contained a homolog for *hasR*, the outer membrane receptor for hemophore, which is essential for heme acquisition (Wandersman and Delepelaire, 2004; Figure 3 and Supplementary Table S4). It has been suggested that bacteria might acquire heme from other organisms (Wandersman and Stojiljkovic, 2000), and our data seem to indicate that R76 and S35 might be able to do so. In the case of R32 and S35, the above-mentioned genes involved in siderophore production and siderophore and hemophore utilization might contribute to their colonizing ability. Numerous studies have demonstrated that mutants in siderophore synthesis and uptake resulted in reduced fitness in the rhizosphere, in the spermosphere and in the phyllosphere (Mirleau et al., 2000; Kahnert et al., 2002; Molina et al., 2005; Drehe et al., 2018).

Comparison of S49 to the closely related R84, for which an endophytic lifestyle was not observed in our assay, revealed the presence of an additional type VI secretion system (T6SS) of subtype 4A in S49, found in none of the other strains (Figures 1, 3). T6SS are involved in competition and predation between bacteria, and thus may play an important role in the competition with other microbiome members for entry sites into the plant (Ho et al., 2014; Bernal et al., 2017). Moreover, it was recently suggested that the biological relevance of the T6SS is not restricted to bacteria-bacteria interactions but encompasses further relevant aspects of biocontrol activity of plant-associated *Pseudomonas* (Vacheron et al., 2019). Expression of T6SS genes during root colonization was reported for *Pseudomonas fluorescens* Pf29Arp and the expression was upregulated when the plant was attacked by the fungus *Gaeumannomyces graminis* var. *tritici* (Barret et al., 2009; Marchi et al., 2013). Additionally, S49 carried genes coding for a S-pyocin of type S6/S7, which were absent from R84 (Figure 3). The production of such bacteriocins can confer advantages for competition and niche colonization. In plant-colonizing *Pseudomonas*, different types of bacteriocins distinguishable by their structure and activity have been described, including the large phage-like R-type tailocins, the modular S-type pyocins, B-type microcins and lectin-like bacteriocins (Ghequire and De Mot, 2014; Jamet and Nassif, 2015; Ghequire et al., 2018). In plant-associated *Pseudomonas*, R-type tailocins are preferably located in a region between the stress response genes *mutS* and *cinA* (Loper et al., 2012; Ghequire et al., 2015). Interestingly, only two genes, a transcriptional regulator and a R2 holin pyocin, were found for most phyllosphere strains in this region (S04, S19, S34, and S35, Supplementary Table S4). In contrast, strain S49 showed a different composition in this genomic region: three hypothetical proteins of unknown function (PspS49_06155, PspS49_06160, PspS49_06165) and a DNA binding protein (PspS49_06150) preceded the transcriptional regulator and the phage tail-like region contained a hydrolase (PspS49_06230) and a hydroxyacid dehydrogenase (PspS49_06310). Such enzymes were also reported in the phyllosphere epiphyte *P. fluorescens* A506 (Ghequire and De Mot, 2014) but their putative involvement in plant colonization is yet to be investigated. Differential competition abilities might be attributable to secretion systems and bacteriocin production, both involved in bacteria-bacteria interactions and competition (Bernal et al., 2017, 2018). The rhizosphere *Pseudomonas* strains differed in the structure and size of the same genomic regions, spanning 16 Kbp in R32, 34 Kbp in R47 and R84, and even 75 Kbp in R76, which is to our knowledge the largest *mutS*/*cinA* region described for plant-associated *Pseudomonas* strain (Supplementary Table S4). Similarly, homologs for the filamentous hemagglutinin *hecA* adhesin gene, involved in the attachment and aggregation of plant pathogens (*Erwinia* spp.), were identified in the four rhizosphere strains R32, R47, R76, R84 and in one phyllosphere strain S49 (Rojas et al., 2002). The T6SS of subtype 4A, the pycocins and the filamentous hemagglutinin might therefore contribute to the competitiveness of S49 and to its ability to colonize plants endophytically.

While R47 did not stand out as a successful endophytical colonizer in our experiment, it successfully colonized Lady Claire plants epiphytically (Figure 7A). In a previous pot study on potato involving R47, R32 and R76, R47 proved to be a highly competitive rhizosphere colonizer. R47 inoculated pots were placed in the same trays as non-inoculated pots. After watering, R47 was re-isolated in high abundance in the uninoculated pots (Guyer et al., 2015). R47 putatively distinguished itself from R32 and R76 in siderophore production. A complete gene cluster for the biosynthesis (*acsABCDEF* + *yhcA*) and transport (*cbrABCD*) of the siderophore achromobactin was found in the genome of R47 (Figure 3). In the plant pathogenic *Erwinia chrysanthemi*, achromobactin was postulated to play a role in the endophytical phase to cope with limited iron availability in the plant (Franza et al., 2005). Its detection in plant beneficial and plant growth promoting *Pseudomonas* endophytes corroborates these findings, suggesting an important role of achromobactin in endophytical colonization of beneficial *Pseudomona*s too (Zhao et al., 2013; Mehnaz et al., 2014; Garrido-Sanz et al., 2016). Genes for the biosynthesis of an exopolysaccharide, Psl, believed to be involved in surface attachment and biofilm formation (Upadhyay et al., 2017), were also exclusively found in R47, although incomplete gene clusters were found in R76 and S35. In both strains, a homolog for *pslD*, involved in the export of Psl seemed however to be lacking (Figure 3 and Supplementary Table S4).

**FIGURE 8:**
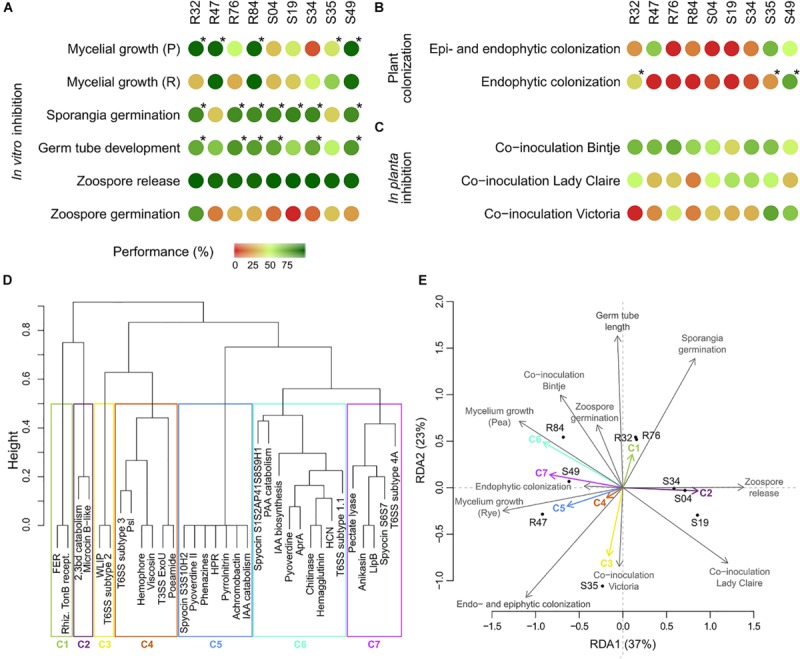
Phenotypical table summarizing the results obtained in the **(A)** *in vitro* bioassays, **(B)** plant colonization assays and **(C)** *in planta* inhibition assays. Inhibition or colonizing abilities were translated to a value of performance, by computing the percentages of corresponding controls (see section Materials and Methods) with maximum inhibition or maximum colonization corresponding to 100%. Mycelial growth (Pea), sporangia germination, germ tube germination and colonization were considered for correlation analysis between accessory genes and phenotype. Stars indicate the strains considered as active. **(D)** Clustering of known antimicrobial compounds for RDA analysis (FER, ferric enterobactin receptor; 2,3bd, 2,3-butanediol; rhiz. TonB recept., rhizobactin TonB receptor; WLIP, white-line-producing; PAA, phenylacetic acid; IAA, indole-3-acetic acid). **(E)** RDA of the results obtained in the bioassays (response variables) and the known genetic determinants table (explanatory variables).

In addition to classical biocontrol approaches relying on one particular organism, we have recently investigated the protection potential of dual and triple combinations of *Pseudomonas* strains on three potato cultivars (De Vrieze et al., 2018). When we assessed the survival rates of R32, R47, S19, S35, and S49 when co-incubated in double and triple combinations, we noticed that S49 appeared to be a good partner to all strains, except to R47, by which it was largely outcompeted. Interestingly, the comparative genomics analysis carried out here revealed that S49 and R47 both harbored a T6SS of subtype 1.1, and that both strains produced three S-pyocins of which two were of the same type. In a study on *P. aeruginosa* and *Vibrio cholerae*, it was demonstrated that the presence of a T6SS in *V. cholerae* triggered the expression and activation of T6SS in *P. aeruginosa*, which led to rapid death of *V. cholerae* cells. This phenomenon, for which the term “T6SS dueling” was proposed (Basler and Mekalanos, 2012; Basler et al., 2014), might well explain the lack of competitiveness of the otherwise very potent competitor S49 toward the similarly equipped R47. R32, like S35, was also often weaker than its competitors and is the only other strain that did not produce any S-pyocin. In the same study, we assessed the strains’ antagonistic potential against *P. infestans* by means of leaf discs of three potato cultivars (De Vrieze et al., 2018). The obtained data, which is summarized in Figure 8C, revealed that strains performing well on Lady Claire were mostly poorly inhibiting *P. infestans* on Bintje, whereas little inhibition was generally observed on Victoria. Overall, S35 was the only strain significantly inhibiting *P. infestans* on leaf discs of all three cultivars. Mining for genes only identified in S35, we identified the CLP WLIP and a T6SS of subtype 2 described above. Both might not only be involved in bacteria-bacteria competition, but also in successful establishment of the strain on plant surfaces, ultimately leading to *in planta* anti-*Phytophthora* activity by this strain, which otherwise was a rather poor *P. infestans* inhibitor in *in vitro* assays (Figure 8 and De Vrieze et al., 2018).

The performance of the different strains in all phenotypic tests described above are summarized in Figures 8A–C. For the colonization assays, only the results for Lady Claire were retained, as colonization was overall more successful on this cultivar. In order to link these differential performances with specific genes or groups of co-occurring genes (Figure 8D and Supplementary Figure S2A), a redundancy analysis on the strains’ phenotypes and their genetic inventories was performed (Figure 8E). Partitioning of variances showed much higher proportion of constrained variance (0.97) than unconstrained variance (0.03), suggesting that a large amount of the variation observed for the phenotypical data might be attributable to the genetic inventories. The analysis illustrated that R32, R47, R76, R84, and S49 showed strong anti-oomycete potential although their individual performances varied considerably across the *in vitro* assays (Figure 8E and Supplementary Figure S2B). The antimicrobial compounds and secretion systems comprised in cluster 5, 6, and 7 are potential genetic determinants for the observed activities on mycelial growth but are unlikely to account for the effects on sporangia germination. The opposite is true for the antimicrobial compounds of cluster 1. Strains S34, S04, and S19 were overall only mildly active against *P. infestans*, which might be explained by their not harboring genes contained in clusters 1, 4, 5, 6, and 7. Strain S35 strongly diverged from the other strains, being particularly performant in the *in planta* inhibition assays (Figure 8C), which could be linked to the genes contained in cluster 3, but showing rather weak anti-*Phytophthora* activity *in vitro*, potentially due to the absence of antimicrobial compounds or secretion systems from clusters 1, 5, 6, and 7. The analysis put forward the compounds in clusters 5 and 6, which showed the overall highest correlations between phenotype and genetic inventory, as the most likely to have anti-*Phytophthora* activity. Both these clusters contain one antimicrobial compound for which anti-*Phytophthora* activity has been described, namely HCN and phenazines, but also others whose putative efficiency against the oomycete remains to be investigated. Likewise, we also observed a positive correlation between the presence of genes in cluster 1 with sporangia germination and germ tube development and to a lesser extent with zoospore germination; in cluster 3 with inhibition on Victoria and with and epi- and endophytic colonization; and in cluster 7 with mycelial growth in both media. These genes therefore represent promising candidates and their involvement in the different phenotypes pertaining to efficient biocontrol of late blight by *Pseudomonas* strains shall be investigated in future studies, e.g., through targeted mutagenesis.

### Searching for New Genetic Determinants of Anti-oomycete Activity

In addition to mining genes previously shown to be involved in either antimicrobial activity or plant colonization (discussed in the section above), we also explored whether we could identify new genetic determinants of anti-*Phytophthora* activity. To this end, we performed a pangenome correlation analysis between (i) the accessory genes shared by different strains, and (ii) the detailed phenotypic data acquired (Figures 4–7), which we used to build different groups of active vs. non-active strains (Figure 8). A first group was constructed based on the plant colonizing ability of the strains and was composed of R32, S35, and S49, which showed an active phenotype. Although we did not find any gene exclusively present in these three strains, four genes were shared by the three active strains and R47, which was a good epiphyte colonizer of Lady Claire (Figure 7). These genes included an outer membrane autotransporter barrel and three adjacent genes coding for a diguanylate cyclase phosphodiesterase, a peroxidase and a histidine kinase (Supplementary Table S5). Diguanylate cyclases are involved in the biosynthesis of cyclic-di-GMP, a major global messenger in bacteria, that acts as a sensory mechanism allowing to adjust the bacterial physiology in reaction to environmental cues, e.g., through biofilm formation, motility and many other processes (Ryan et al., 2006). A blast search revealed the presence of the two domains responsible for synthesis and degradation of c-di-GMP respectively. It was reported for *P. fluorescens* that some diguanylate cyclases were promoting biofilm formation, e.g., through regulation of adhesin (LapA) and swimming motility (Newell et al., 2011). Increased ability to form biofilms might indeed be an important element in the better colonization capacity displayed by these strains, both endophytically and epiphytically.

In terms of direct anti-oomycete activity, seven out of nine strains actively inhibited sporangia germination (all but R47 and S35), as summarized in Figure 8. A single gene coding for a lipoprotein was present exclusively in all active strains, suggesting its putative involvement in the inhibition of *P. infestans* sporangia germination. We did not find any gene common to the group formed by R32, R76, R84, S04, S34, and S49, which displayed similar inhibitory activity on germ tube development. However, two genes, a peptide synthase and a cytosine purine, uracil, thiamine, allantoin permease were found in all strains but S04 (Supplementary Table S5).

**FIGURE 9:**
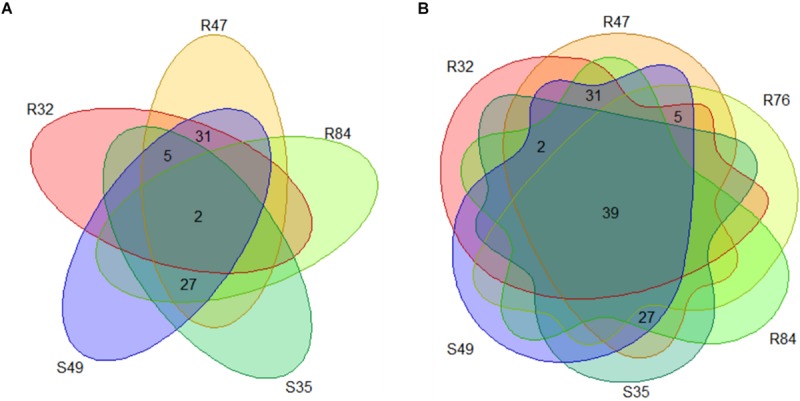
**(A)** Number of genes positively correlated to mycelial growth inhibition and exclusively present in *Pseudomonas* strains with activity on mycelial growth. **(B)** Number of genes positively correlated to mycelial growth inhibition present in the active strains and R76.

**FIGURE 10:**
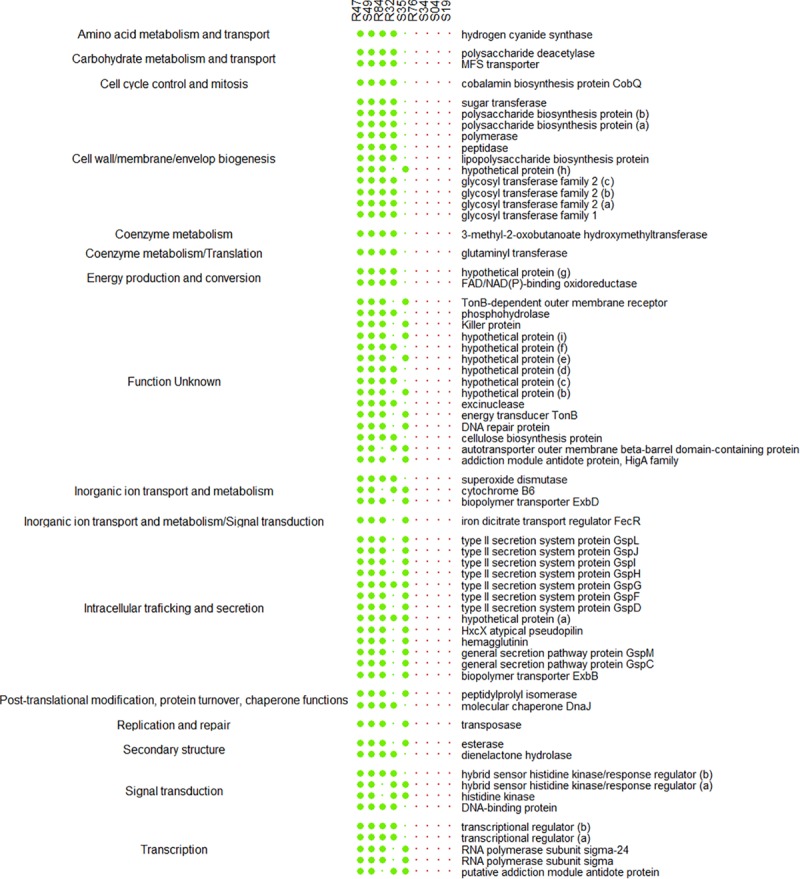
List of the genes positively correlated to mycelial growth inhibition and exclusively present in at least four *Pseudomonas* strains with activity on mycelial growth. Genes are sorted according to their functional COG category.

**FIGURE 11:**
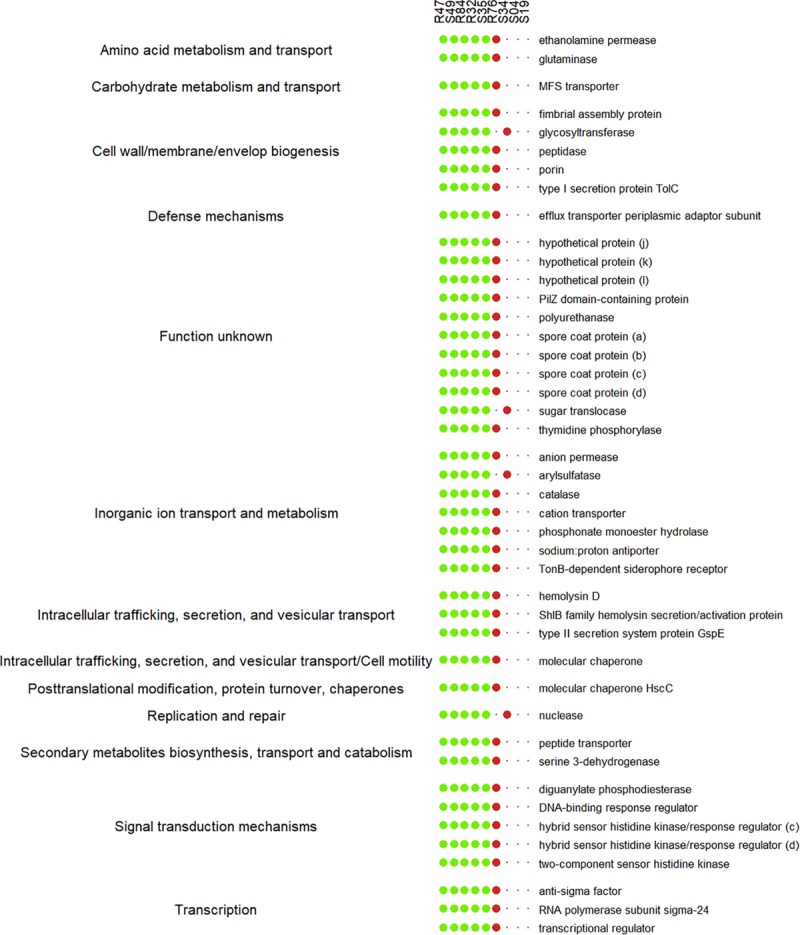
List of genes positively correlated to mycelial growth inhibition present in the active strains and R76 or S34. Genes are sorted according to their functional COG category.

A last group of strains was formed based on the *in vitro* mycelium-inhibiting activity and included R32, R47, R84, S35, and S49. Here, a substantial set of genes were present in a subset of the strains sharing this phenotype (Figure 9A and Supplementary Table S5). A list of these genes correlating with the active phenotype is given in Figure 10. Two genes were common and exclusively present in all active strains, one hypothetical protein putatively involved in intracellular trafficking and secretion and a gene encoding a Type II secretion system (T2SS) protein, GspG (Figure 10). Interestingly, we observed that several genes were common to all active strains, but also shared with R76, while they were absent from all other inactive strains (Figures 9B, 11). Among these genes are two putative peptidases (PspR32_03440, PspR32_00585), the first belonging to the TolC superfamily of outer membrane proteins of the type I secretion system and flanked by a gene putatively coding for hemolysin D (PspR32_03435). A diguanylate phosphodiesterase (PspR32_06500) flanked by two histidine kinase response regulators, a putative fimbrial cluster and a putative porin and its associated type I secretion system (PspR32_20390 to PspR32_20415) were also found (Figure 11). In contrast, we found 31 genes shared by the four strongest mycelial inhibiting strains R32, R47, R84, and S49, but not S35. As expected, these genes included *hcnB* and *hcnC*, involved in the biosynthesis of the volatile hydrogen cyanide (Figure 10). A cluster of genes containing a putative cellulose biosynthesis protein and a putative cobalamin biosynthesis protein were also among these 31 genes. Cellulose produced by *P. fluorescens* SBW25 was shown to be essential for biofilm formation at the interface between air and liquid (Spiers et al., 2003). *sodC*, coding for a copper zinc superoxide dismutase and thought to be involved in bacterial defense against extracellular reactive oxygen species was identified too (Imlay and Imlay, 1996). Furthermore, 27 genes shared by all strains except for R32 were positively correlated to mycelial growth inhibition (Figures 9A, 10). The analysis highlighted genes coding for a cell surface signaling system for the use of ferric dicitrate; a T2SS containing the above-mentioned *gspG*; filamentous hemagglutinin; a putative porin coupled to a TonB-ExbB-ExbD complex; a hypothetical protein containing a hemolysin activation/secretion protein domain and a putative antitoxin (a transposase); a HigA/HigB like toxin/antitoxin system and a PpiC-type peptidylprolyl isomerase (Figure 10). In R84, S35, and S49, a gene coding for alkaline phosphatase was found adjacent to the T2SS gene cluster, while for R47 two hypothetical proteins containing phosphate-binding domains were found. In *P. aeruginosa*, the toxin/antitoxin system *higB/higA* is widespread and was shown to negatively impact the production of pyochelin, swarming and biofilm formation (Wood and Wood, 2016). Interestingly, peptidylprolyl isomerase was reported to induce resistance against plant pathogens in tobacco plants (Shumilina et al., 2006).

## Conclusion and Perspectives

In conclusion, *de novo* sequencing, genome assembly and phylogenetic analysis revealed that eight of the nine studied strains belonged to the *Pseudomonas fluorescens* group. Strain R47 clustered closely to famous antagonistic *P. chlororaphis* and *P. protegens* strains and encoded the highest number of known genetic determinants of antagonism in terms of antibiotics and toxins. Still, it was outperformed by many of the other strains in inhibiting sporangia and zoospore germination suggesting the presence of many yet unknown anti-oomycete determinants. Despite having the smallest genome, R32 was the strongest inhibitor of *P. infestans* across all *in vitro* assays and appeared to be a competent plant colonizer as well. In contrast to all other strains, R32 clustered in the *P. putida* subgroup. While *P. putida* species have been extensively studied primarily for their potential use in bioremediation and secondarily for their antagonistic activities, it is likely that these strains carry additional genes yet to be described and linked to antagonistic traits in comparison with the more widely investigated fluorescent *Pseudomonas*. Beyond these two rhizosphere strains, two phyllosphere isolates were remarkable in being either able to best colonize different potato cultivars endophytically (S49) or to best protect them against *P. infestans* infection (S35). When designating the most promising strains against potato late blight, the differential abilities of the strains to affect different steps of the pathogen’s lifecycle should be taken into account. If the antagonists are intended for preventive use, one should privilege strains active against sporangia germination with good colonizing abilities, such as S49 and, according to our previous data, strain S35. To mitigate the onset and spreading of an existing infection, strains R32, R47, R84, and S49 might be the most interesting biocontrol candidates, because of their overall strong anti-*Phytophthora* activity and their drastic effects on mycelial growth. If the antagonist is to prevent zoospore release from an established infection and thereby avoid infection of daughter tubers, as is often required in the second half of the potato growing season, all nine strains are promising candidates.

Comparative analysis of the strains’ phenotypic features and of their comprehensive gene inventories suggested the putative involvement of multiple compounds and mechanisms in the inhibition of *P. infestans*, in plant colonization and in competition with other microbes. In particular, our analysis identified several candidates of interest to explain both the anti-*Phytophthora* activity and the potato colonizing ability of the strains. These include among others HCN, enzymes involved in chitin and protein degradation as well as in cellulose biosynthesis, cyclic lipopeptides, a lipoprotein potentially functioning as an auxiliary component of an ABC transporter, exopolysaccharides, siderophores, bacteriocins and other toxins, as well as different secretion systems. Future work involving both global transcriptomic and targeted mutagenesis approaches will help to identify the most relevant genes and to quantify their relative contribution to the observed phenotypes. This will bring significant advance in unraveling the complex genetic basis of *Pseudomonas-*mediated plant health protection, with the ultimate goal to develop more efficient and reliable biocontrol strategies against late blight and other major plant diseases.

## Data Availability Statement

The datasets generated for this study can be found in the NCBI, CP019396, CP019399, CP019428, CP019426, CP019427, CP019397, CP019398, CP019431, CP019432.

## Author Contributions

LW, CA, AV, MD, and AB designed research. MD performed the bioassays. AV performed genome annotation, comparative genomics and correlation analysis with help from KS. MD, AV, and RR analyzed the data. MD and AV wrote the manuscript with help from LW, CA, RR, and AB.

## Conflict of Interest

The authors declare that the research was conducted in the absence of any commercial or financial relationships that could be construed as a potential conflict of interest.

## Footnotes

1 http://tree.bio.ed.ac.uk/software/figtree
