# Supplementary material for: Linking Comparative Genomics of Nine Potato-Associated Pseudomonas Isolates With Their Differing Biocontrol Potential Against Late Blight
Source: Front Microbiol. 2020 Apr 30;11:857. doi: 10.3389/fmicb.2020.00857 (PMC7204214; doi:10.3389/fmicb.2020.00857)
Supplement: Supplementary file 1 [file Data_Sheet_1.docx]

**Supplementary material**

The Supplement contains 2 supplementary figures and 5 supplementary tables.

**Figures**

**
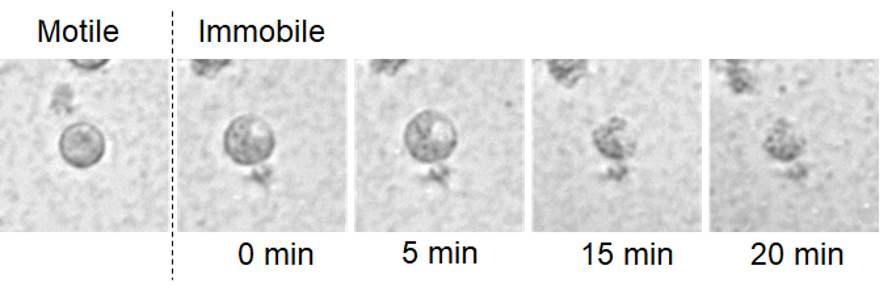
**

**Figure S1** Time lapse images of the immobilization and degradation of a zoospore exposed to R32 in a bacterial suspension. Pictures were taken every 5 minutes using a Cytation5 plate reader (Biotek, United States).


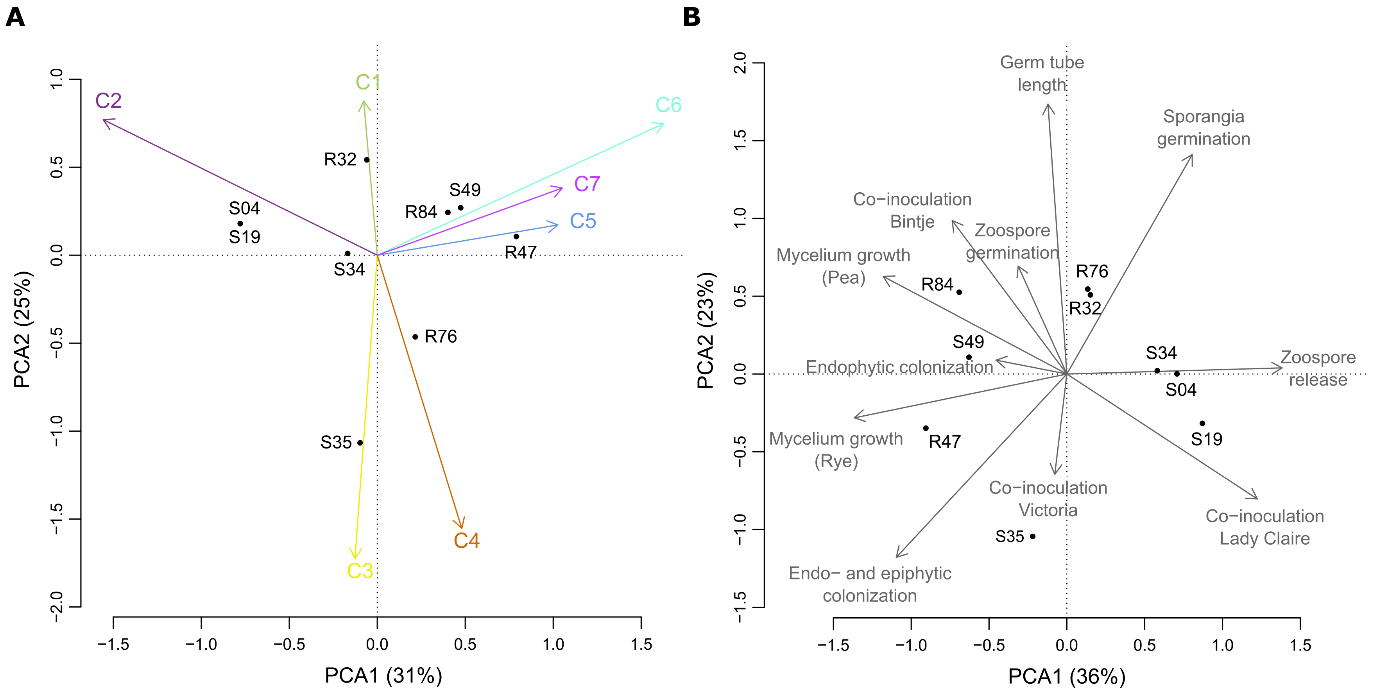


**Figure S2** (A) PCA analysis on the clusters of co-occurring genes. (B) PCA analysis on the phenotypic data of the *in vitro* experiments, plant colonization experiments and co-inoculation experiments.

**Tables**

**Table S1:** NCBI data submission information. Bioproject, Biosample, SRA and Genbank accession are given for each strain.

| **Strain** | **Bioproject** | **Biosample** | **SRA acc.#** | **GenBank acc. #** |
| --- | --- | --- | --- | --- |
| PspR32 | PRJNA355625 | SAMN06234845 | SRS1925888 | CP019396 |
| PchlR47 | PRJNA355625 | SAMN06241861 | SRS1929209 | CP019399 |
| PspR76 | PRJNA355625 | SAMN06242059 | SRS1932041 | CP019428 |
| PspR84 | PRJNA355625 | SAMN06242059 | SRS1932279 | CP019426 |
| PspS04 | PRJNA355625 | SAMN06246385 | SRS1932352 | CP019427 |
| PspS19 | PRJNA355625 | SAMN06234846 | SRS1925892 | CP019397 |
| PspS34 | PRJNA355625 | SAMN06241450 | SRS1929109 | CP019398 |
| PspS35 | PRJNA355625 | SAMN06241957 | SRS1932016 | CP019431 |
| PspS49 | PRJNA355625 | SAMN06245871 | SRS1932214 | CP019432 |

**Table S2**: Strains used for construction of the phylogenetic tree and their accession numbers

| **No.** | **Strain** | **Accession** |
| --- | --- | --- |
| 1. | *Pseudomonas aeruginosa* PA7 | NC_009656 |
| 2. | *Pseudomonas aeruginosa* PACS2 | NZ_AAQW01000001 |
| 3. | *Pseudomonas aeruginosa* PAO1 | NC_002516 |
| 4. | *Pseudomonas alkylphenolia* strain KL28 | CP009048 |
| 5. | *Pseudomonas antarctica* strain PAMC 27494 | CP015600 |
| 6. | *Pseudomonas azotoformans* strain S4 | CP014546 |
| 7. | *Pseudomonas brassicacearum* subsp. brassicacearum NFM421 | NC_015379 |
| 8. | *Pseudomonas brenneri strain* BS2771 | NZ_LT629800 |
| 9. | *Pseudomonas chlororaphis* subsp. aurantiaca strain JD37 | NZ_CP009290 |
| 10. | *Pseudomonas chlororaphis* subsp. aureofaciens 30-84 | NZ_CM001559 |
| 11. | *Pseudomonas chlororaphis* O6 | NZ_CM001490 |
| 12. | *Pseudomonas chlororaphis* strain PA23 | NZ_CP008696 |
| 13. | *Pseudomonas chlororaphis* strain PCL1606 | NZ_CP011110 |
| 14. | *Pseudomonas corrugata* strain RM1-1-4 | NZ_CP014262 |
| 15. | *Pseudomonas fluorescens* A506 | NC_017911 |
| 16. | *Pseudomonas fluorescens* BBc6R8 | NZ_AKXH00000000 |
| 17. | *Pseudomonas fluorescens* strain DSM 50090 | LHVP01000000 |
| 18. | *Pseudomonas fluorescens* F113 | CP003150 |
| 19. | *Pseudomonas fluorescens* Pf0-1 | NC_007492 |
| 20. | *Pseudomonas fluorescens* SBW25 | AM181176 |
| 21. | *Pseudomonas fluorescens* SS101 | NZ_CM001513 |
| 22. | *Pseudomonas fluorescens* strain UK4 | NZ_CP008896 |
| 23. | *Pseudomonas granadensis* strain LMG 27940 | LT629778 |
| 24. | *Pseudomonas koreensis* strain D26 | CP014947 |
| 25. | *Pseudomonas lini* strain BS3782 | LT629746 |
| 26. | *Pseudomonas mandelii* 36MFCvi | NZ_KB906353 |
| 27. | *Pseudomonas mandelii* JR-1 | CP005960 |
| 28. | *Pseudomonas moraviensis* strain BS3668 | LT629788 |
| 29. | *Pseudomonas poae* RE*1-1-14 | NC_020209 |
| 30. | *Pseudomonas protegens* CHA0 | CP003190 |
| 31. | *Pseudomonas protegens* Pf-5 | NC_004129 |
| 32. | *Pseudomonas psychrophila* strain BS3667 | NZ_LT629795 |
| 33. | *Pseudomonas putida* BIRD-1 | NC_017530 |
| 34. | *Pseudomonas putida* H8234 | NC_021491 |
| 35. | *Pseudomonas putida* KT2440 | NC_002947 |
| 36. | *Pseudomonas putida* ND6 | NC_017986 |
| 37. | *Pseudomonas putida* S16 | NC_015733 |
| 38. | *Pseudomonas putida* W619 | NC_010501 |
| 39. | *Pseudomonas reinekei* strain BS3776 | NZ_LT629709 |
| 40. | *Pseudomonas sp.* 20_BN | NZ_CCSF01000001 |
| 41. | *Pseudomonas sp.* A3 | CP014870 |
| 42. | *Pseudomonas sp.* CMR5c | NZ_LHUY00000000 |
| 43. | *Pseudomonas sp.* UW4 | NC_019670 |
| 44. | *Pseudomonas sp.* Z003-0.4C(8344-21) | LT629756 |
| 45. | *Pseudomonas stutzeri* A1501 | NC_009434 |
| 46. | *Pseudomonas syringae* pv. syringae B728a | NC_007005 |
| 47. | *Pseudomonas syringae* pv. tomato str. DC3000 | NC_004578 |
| 48. | *Pseudomonas veronii* 1YdBTEX2 | LT599583 |
| 49. | *Azotobacter vinelandii* DJ | NC_012560.1 |

**Tables S3 to S5, please see separate Excel files**

**Table S3**: The list of genes from each of the nine genomes including their annotation obtained from NCBI, and COG categories, additional functional annotation information from interproscan and eggnog.

**Table S4**: The first table contains to the list of genes and corresponding reference protein sequences and locus_tags of the homologs found in the nine *Pseudomonas* strains. The reference protein sequences were searched against the genomes of the nine *Pseudomonas* strains using blastp (v 2.2.30+) (Altschul *et al.*, 1990). Only the best matching hit satisfying certain threshold parameters (e-value ≤ 1e-5, percentage identity ≥ 50% and query coverage ≥ 50%) was taken as homolog in each of the nine *Pseudomonas* strains. The second table contains the genes belonging to putative R-pyocins found between the *mutS* and *cinA* genes for each strain.

**Table S5**: List of genes and corresponding locus tags positively correlated to mycelial growth inhibition, plant colonization and inhibition of sporangia germination. For mycelial growth inhibition, all genes present in at least four of the five active strains (R32, R47, R84, S35 and S49) or present in all five active strains and R76 or S34 are listed.

**Reference**

Altschul, S.F., Gish, W., Miller, W., Myers, E.W., and Lipman, D.J. (1990) Basic local alignment search tool. *J. Mol. Biol.* **215**: 403–410.
